# Generation of Naïve Bovine Induced Pluripotent Stem Cells Using PiggyBac Transposition of Doxycycline-Inducible Transcription Factors

**DOI:** 10.1371/journal.pone.0135403

**Published:** 2015-08-19

**Authors:** Takamasa Kawaguchi, Tomoyuki Tsukiyama, Koji Kimura, Shuichi Matsuyama, Naojiro Minami, Masayasu Yamada, Hiroshi Imai

**Affiliations:** 1 Laboratory of Reproductive Biology, Graduate School of Agricuture, Kyoto University, Kyoto, Japan; 2 Research Center for Animal Life Science, Shiga University of Medical Science, Shiga, Japan; 3 Animal Feeding and Management Research Division, NARO Institute of Livestock and Grassland Science, Tochigi, Japan; Institute of Medical Biology, SINGAPORE

## Abstract

Generation of pluripotent stem cells (PSCs) in large domestic animals has achieved only limited success; most of the PSCs obtained to date have been classified as primed PSCs, which possess very little capacity to produce chimeric offspring. By contrast, mouse PSCs have been classified as naïve PSCs that can contribute to most of the tissues of chimeras, including germ cells. Here, we describe the generation of two different types of bovine induced pluripotent stem cells (biPSCs) from amnion cells, achieved through introduction of piggyBac vectors containing doxycycline-inducible transcription factors (*Oct3/4*, *Sox2*, *Klf4*, and *c-Myc*). One type of biPSCs, cultured in medium supplemented with knockout serum replacement (KSR), FGF2, and bovine leukemia inhibitory factor (bLIF), had a flattened morphology like human PSCs; these were classified as primed-type. The other type biPSCs, cultured in KSR, bLIF, Mek/Erk inhibitor, GSK3 inhibitor and forskolin, had a compact morphology like mouse PSCs; these were classified as naïve-type. Cells could easily be switched between these two types of biPSCs by changing the culture conditions. Both types of biPSCs had strong alkaline phosphatase activity, expressed pluripotent markers (*OCT3/4*, *NANOG*, *REX1*, *ESRRβ*, *STELLA*, and *SOCS3*), and formed embryoid bodies that gave rise to differentiated cells from all three embryonic germ layers. However, only naïve-type biPSCs showed the hallmarks of naïve mouse PSCs, such as LIF-dependent proliferation, lack of FGF5 expression, and active *XIST* expression with two active X chromosomes. Furthermore, naïve-type biPSCs could contribute to the inner cell mass (ICM) of host blastocysts and most tissues within chimeric embryos. This is the first report of generation of biPSCs with several characteristics similar to those of naïve mouse PSCs and a demonstrated potential to contribute to chimeras.

## Introduction

Somatic cells can be reprogrammed to a pluripotent state via ectopic expression of the transcription factors Oct4, Sox2, Klf4, and c-Myc, thereby generating induced pluripotent stem cells (iPSCs) [1, 2]. Pluripotent stem cells (PSCs), including iPSCs and embryonic stem cells (ESCs), can be classified into two categories: ‘naïve’ and ‘primed’ PSCs [3–5]. Naïve PSCs correspond to ICM of blastocysts, and are similar to mouse ES cells (mESCs), whereas primed PSCs correspond to the epiblast at the postimplantation stage, as represented by mouse epiblast stem cells (mEpiSCs) and human ES cells (hESCs). Naïve PSCs exhibit some distinctive characteristics, such as a compact and dome-shaped morphology, the ability to be passaged as a single cell, dependence of proliferation on the leukemia inhibitory factor (LIF)–Jak/Stat signaling pathway, two active X chromosomes (X_a_X_a_), and specific expression of REX1, ESRRβ, and STELLA [3, 4]. In addition, naïve PSCs are more frequent in homologous recombination [6], and more efficient in repopulating the ICM region after aggregation or injection into host blastocysts; this feature allows them to develop into chimeras that, in turn, can transmit mutated or modified genes to subsequent generations via the germline [3, 7]. On the other hand, primed PSCs have a flattened morphology, basic fibroblast growth factor (bFGF)-dependent proliferation, an inactivated X chromosome (X_a_X_i_), and a relatively limited capacity to produce chimeras [8]. Recent studies demonstrated that primed human PSCs can be converted into a naïve state using certain chemical compounds (a GSK3β inhibitor and a MEK/ERK inhibitor (2i), and the protein kinase A pathway agonist forskolin) [9]. Thus, naïve PSCs are a feasible potential source of material for production of PSC-derived offspring in domestic species.

Naïve iPSCs and PSCs in large animals, such as cattle, could be applied to genetic improvement of domestic animals as well as preclinical models for human regenerative medicine and disease. To date, however, it has been quite difficult to establish naïve iPSCs and PSCs in mammals other than rodents; most of the iPSCs reported in monkeys [10, 11], pigs [12–15], and rabbits [16] have been of the primed type. In cattle, Sumer et al. [17] reported the generation of bovine iPSCs (biPSCs), but these cells exhibited characteristics of primed iPSCs. The major reason for this is that the optimal culture conditions for generation and maintenance of iPSCs in various mammalian species have not been determined. Recently, Tsukiyama et al. [18] proposed an efficient system using piggyBac (PB) transposition of doxycycline (Dox)-inducible transcription factors, which allows the expression of introduced reprogramming factors to be controlled by the presence or absence of Dox, for screening of culture conditions for generation of iPSCs. The PB transposon vector [19–21] is a non-viral and safe vector system, in which integrated constructs (including reprogramming factors) can be removed by the re-expression of transposase (PBase). On the other hand, the source of somatic cells for iPSCs significantly affects reprogramming efficiency [22, 23]. For example, mouse neural progenitor cells can be more efficiently reprogrammed than fibroblasts, and iPSCs can be established from these cells by expression of only one or two exogenous factors, due to their endogenous expression of Sox2 and c-Myc [23]. However, neural progenitor cells are not easily accessible, and are not available in large quantities. By contrast, amnion-derived cells (ADCs) can be readily obtained from the placental tissue after delivery. The amnion, derived from the epiblast as early as the eighth day after fertilization, is a thin membrane-lined cavity filled with fluid that protects the developing fetus. Moreover, in both human and mouse, reprogramming of ADCs into iPSCs is more efficient and faster than that of fibroblasts [24–26]. Therefore, ADCs represent an ideal cell source for the generation of iPSCs.

In this experiment, we attempted to establish biPSCs from bovine amnion-derived cells by introducing Dox-inducible PB vectors expressing the mouse reprogramming factors (*Oct3/4*, *Klf4*, *Sox2*, and *c-Myc*) and culturing cells in the presence of certain chemical compounds (2i and forskolin). Two different types (primed and naïve) of biPSCs appeared under different culture conditions, and we characterized the pluripotent properties of the resultant biPSCs both *in vitro* and *in vivo*.

## Materials and Methods

### Ethics statements

All cattle were fed grass silage-based diet *ad libitum*. All procedures involving the care and use of animals were approved by the Animal Research Committee of NARO institute of Livestock and Grassland Science.

### Isolation of bovine amnion-derived cells (bADCs)

Bovine amnion layer was harvested from a female Japanese black cattle fetus at 50 days of gestation at the National Institute of Livestock and Grassland Science, Japan. The amnion was mechanically peeled away from the chorion and the allantois, divided into small pieces with fine surgical scissors, and dissociated by incubating for 2 hours at 37°C with 0.3% collagenase (Wako) in Dulbecco’s modified Eagle’s medium (DMEM, Invitrogen) containing 10% fetal bovine serum (FBS, JRH Biosciences). After collagenase digestion, the cell suspension was kept at room temperature for 5 min, and then poured through a cell strainer; the filtered suspension was then centrifuged at 200 *g* for 5 min. The precipitated cells were cultured in DMEM (Sigma) containing 10% FBS, penicillin (Sigma), and streptomycin (Sigma). When the cells reached confluence, they were cryopreserved in liquid nitrogen until use.

### Preparation of bovine LIF

Total RNA was isolated from bovine fetal fibroblasts (bFFs) using TRIzol RNA Isolation Reagents (Invitrogen). DNase I (Takara) was added to the RNA preparation to avoid genomic DNA contamination. For reverse transcription, ReverTra Ace (Toyobo) and Random Primer (Invitrogen) were used. Polymerase chain reaction (PCR) was then performed to amplify the bovine LIF (bLIF) cDNA sequence (GenBank accession no. NM_173931.1) using the KOD-Plus- Neo kit (TOYOBO) and specific primers: sense, 5’- GGA GAG CTC CAC CAT GAA GGT CTT GGC GGC AGG -3’; reverse, 5’- AAG GCT AGC CTA GAA GGC CTG GGC CAG CA -3’. The amplified cDNA fragment (630 bp) was inserted into the pCAGGS vector [27]. Specifically, after digestion of the vector with *Sac*I and *Nhe*I (Takara Bio), the cDNA was inserted downstream of the chicken β-actin–based promoter (CAG) in pCAGGS to create the bLIF expression vector. This vector was transfected into COS-7 cells using Lipofectamine LTX (Invitrogen), and the conditioned medium was collected, filtered, and stored at -20°C until use. The conditioned medium was diluted 1:1000 for biPSC culture.

### Culture of bADCs and biPSCs

bADCs were maintained on collagen-coated (Nitta Gelatin) dishes in somatic cell medium consisting of DMEM containing 10% FBS, 50 ng/ml epidermal growth factor (EGF, Calbiochem), penicillin, and streptomycin. The cells were dissociated enzymatically with TrypLE Select (Invitrogen) for further propagation.

Primed- and naïve-type biPSCs were generated from bADCs as described below. Primed-type biPSCs resembling hESCs were maintained in primed iPSC medium (piPSC medium) consisting of Dulbecco's Modified Eagle Medium/Nutrient Mixture F-12 (DMEM/F12, Invitrogen) containing 20% Knockout Serum Replacement (KSR, Invitrogen), 2 mM L-glutamine (MP Biomedicals), 1× MEM nonessential amino acids (NEAA, Invitrogen), 0.1 mM 2-mercaptoethanol (2-ME, Wako), penicillin, and streptomycin supplemented with 2.0 μg ⁄mL doxycycline (Dox, Sigma), 5 ng/mL human basic fibroblast growth factor (bFGF, Wako or ReproCELL), and bLIF-conditioned medium (1:1000 dilution), prepared as described above. Cells were subcultured every 7 days by physical splitting using a pulled Pasteur pipette, and maintained on 35-mm-diameter cell culture dishes (IWAKI) on a feeder layer of 3–5 × 10^5^ cells SNL cells [28, 29] inactivated with mitomycin C (Sigma).

Naïve-type biPSCs were maintained in naïve iPSC medium (niPSC medium), which consisted of piPSC medium supplemented with 1 μM MEK/ERK inhibitor (PD) (PD0325901, REAGENTS DIRECT), 3 μM GSK3β inhibitor (CH) (CHIR99021, REAGENTS DIRECT), and 10 mM forskolin (FK) (REAGENTS DIRECT) in the absence of bFGF. Cells were subcultured every 4 days by enzymatic dissociation using TrypLE Select and maintained on an SNL feeder layer.

Medium lacking bLIF and containing 20 μM JAK Inhibitor I (Calbiochem #420099) was used to test the influence of the LIF signal pathway on self-renewal and survival of biPSCs. Cells were maintained on an SNL feeder layer, and cell number was counted.

All the cultures were maintained in a humidified incubator at 38.5°C with 5% CO_2_ in air.

### Reprogramming of bADCs

**Fig 1:**
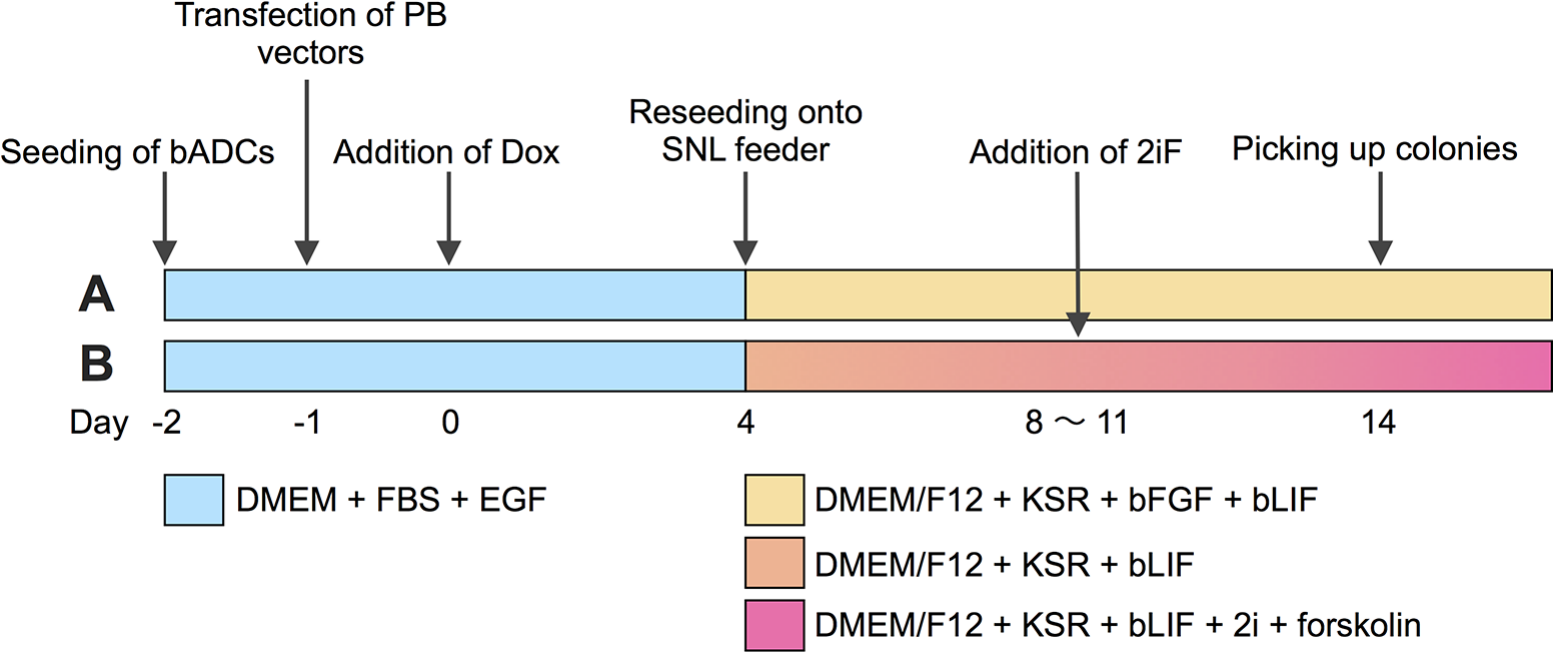
Reprogramming of bovine amnion-derived cells (bADCs) into iPSCs using Dox-inducible PB vectors. (A) Timeline for the establishment of primed-type biPSC lines. (B) Timeline for the establishment of naïve-type biPSC lines.

bADCs were plated at 1.25 × 10^5^ cells per 35-mm dish in culture medium without antibiotics and incubated overnight. Cells were then transfected using Lipofectamine LTX. Briefly, equal amounts (0.4 μg) of hyPBase vector (pCAG-hyPBase [30]), PB vectors with reprogramming factors (PB-TET-OKS and pPB-TET-c-Myc [18, 20, 31]), rtTA PB vector (PB-CAG-rtTA Adv, Addgene), and/or TagRFP PB vector (pPBCAG-TagRFP-IH [18]), 2 μL of Plus reagent (Invitrogen), and 10 μL of Lipofectamine LTX transfection reagent were diluted and mixed in 400 μL of Opti-MEM medium (Invitrogen). DNA–lipid complex was then added to the culture dish. The culture medium was changed 6 hours after transfection. One day after transfection, the culture was supplemented with 2.0 μg ⁄mL Dox. Four days after Dox addition, cells were dissociated with TrypLE Select, 1 × 10^5^ cells were reseeded on an SNL feeder layer, and the medium was replaced with piPSC or niPSC medium lacking 2i and forskolin. Eight days after the addition of Dox, 2i and forskolin were added to niPSC medium. Fourteen days after the addition of Dox, primary colonies were mechanically picked and isolated (in the case of primed-type biPSCs) or chemically dissociated (in the case of naïve-type biPSCs) and transferred onto an SNL feeder layer in 48-well or 4-well plates. The medium was changed every 1–2 days, depending on cell growth (Fig 1).

The original vectors (PB-TET-MKOS, PB-CAG-rtTA Adv, and pCX-OKS-2A) were obtained from Addgene (plasmids 20959, 20910, and 19771, respectively [20, 31]). The empty PB vector and the c-Myc PB vector (pPB-TET-c-Myc) were kind gifts from Dr. Hitoshi Niwa at the RIKEN Center for Developmental Biology. The hyPBase vector (pCMV-hyPBase) was a kind gift from Dr. Keisuke Yusa at Sanger Institute. To generate pCAG-hyPBase, pCMV-hyPBase was cloned into blunt-ended pCAGGS.

### Alkaline phosphatase and immunofluorescence staining

Alkaline phosphatase staining was performed using the Vector Alkaline Phosphatase Substrate kit (Vector). For immunofluorescence analysis, cells were fixed with PBS containing 3.7% paraformaldehyde for 10 min at room temperature. After washing with PBS, cells were blocked with 5% bovine serum albumin (Sigma) and 0.1% Triton X-100 (Sigma) for 45 min at room temperature, and then incubated overnight at 4°C with primary antibodies against OCT3/4 (1:25, SC-5279, Santa Cruz Biotechnology), NANOG (1:250, AB5731, Millipore), glial fibrillary acidic protein (GFAP, 1:100, Z0334, DAKO), actin smooth muscle (ASM, 1:1000, MS-113-P0, Thermo), or alpha-fetoprotein (AFP, 1:100, MAB1368, R&D Systems). Alexa Fluor 594 goat anti-mouse IgG or IgM (1:500, Invitrogen), Alexa Fluor 594 goat anti-rabbit IgG (1:500, Invitrogen), Alexa Fluor 488 goat anti-mouse IgG or IgM (1:500, Invitrogen), and Alexa Fluor 488 goat anti-rabbit IgG (1:500, Invitrogen) were used as secondary antibodies. Nuclei were stained with 1 μg ⁄mL Hoechst 33342 (Sigma).

### Reverse transcription PCR

Total RNAs of cells were prepared using TRIzol reagent. DNase I was added to preparations to avoid genomic DNA contamination. For reverse transcription, ReverTra Ace and Random Primer were used. PCR was carried out with ExTaq (Takara). An example PCR condition was as follows: 94°C for 2 min, followed by 35 amplification cycles (94°C, 20 s; 60°C, 30 s; 72°C, 30 s). The reaction was terminated by an elongation step at 72°C for 7 min. Primer sequences are shown in S1 Table.

### Differentiation of biPSCs *in vitro*

To produce embryoid bodies (EBs), established biPSCs were harvested by trypsinization (in case of naïve-type biPSCs) or physical splitting (in case of primed-type biPSCs), and then transferred to MPC-treated round-bottom dishes (Nunc) in Iscove’s modified Dulbecco’s medium (IMDM, Invitrogen) containing 15% FBS, 2 mM L-glutamine, 1 mM sodium pyruvate, 1× NEAA, 0.1 mM 2-mercaptoethanol, penicillin, and streptomycin supplemented with 2.0 μg/mL Dox. After 3 days of culture, the medium was changed to fresh medium without Dox. After an additional 3 days of culture, floating cell masses were transferred onto gelatin-coated dishes and cultured in 10% FBS DMEM for another 6 days. The resulting cell culture was analyzed by immunocytochemistry and reverse-transcription PCR, as described above.

### Karyotype analysis

Karyotype analysis of the established biPSC lines (pbiPSCs at 65 passages and nbiPSCs at 10 passages) was performed using KaryoMAX Colcemid Solution (Invitrogen) at the time of subculture. For each cell line, 20 metaphase plates were counted.

### Generation of chimeric fetuses from naïve-type biPSCs

RFP-expressing naïve-type biPSCs were generated for the analysis of chimera production. bADCs were transfected with a constitutive TagRFP-expressing PB vector (pPBCAG-TagRFP-IH) simultaneously with pCAG-hyPBase PB-TET-OKS, pPB-TET-c-Myc, and PB-CAG-rtTA Adv, and RFP-expressing colonies were picked and propagated.

For aggregation of biPSCs with bovine embryos, *in vitro* fertilized host embryos were prepared. *In vitro* maturation, fertilization, and culture were performed as described previously [32]. Sixty hours after fertilization, 8- to 16-cell stage embryos were collected and transferred to acid Tyrode solution (pH 1.5) until the zona pellucida was completely dissolved. Microwells (WOWs; Vajta et al., 2000) were prepared in four-well dishes (Nunc) by making microspots with an aggregation needle (BLS) filled with 50 mL of modified SOF medium containing 1.5 mM glucose and 10% KSR and supplemented with 2.0 μg/mL Dox, bLIF-conditioned medium (1:1000 dilution), 1 μM PD0325901, 3 μM CHIR99021, and 10 mM forskolin, and then covered with 400 μL of paraffin oil. Subsequently, the zona-free 8- to 16-cell stage embryos were transferred into the WOWs, and 20–30 naïve-type biPSCs were also transferred and co-cultured at 38.5°C in an atmosphere consisting of 5% CO_2_, 5% O_2_, and 90% N_2_. Five days after initiation of co-cultivation, the aggregated embryos were collected. Three aggregated embryos were then transplanted into each uterine horn of a Japanese black cow at 7 days after heat. Transplantation was performed using a catheter (Misawa Medical Industry).

Pregnancy was diagnosed by rectal palpation or ultrasonography. At 90 days after transplantation, the pregnant cow was sacrificed by an overdose of sodium pentobarbital; the uteri were isolated, dissected, and rinsed with PBS. Fetuses were then isolated, rinsed with PBS, and separated by individual organs/tissues including the brain, skin, lung, liver, stomach, small intestine, large intestine, heart, kidney, spleen, muscle, gonad, amnion, and placenta. Genomic DNA (gDNA) was isolated from the tissues by using TRIzol reagent. Genomically integrated Oct3/4-2A-Klf4 sequences in these samples was detected by PCR analysis of isolated gDNA using O-2A-K (Tg) primers (S1 Table). PCR products from all tissues were confirmed by DNA sequencing. Frozen tissue sections isolated from the chimera were prepared using OCT compounds, and the tissue sections were immunostained with anti-Vasa (1:200, ab13840, Abcam) and/or anti-RFP (1:500, MM-0172-P, MédiMabs) primary antibodies, and Alexa Fluor 488 goat anti-rabbit IgG (1:500, Invitrogen) and/or Alexa Fluor 594 goat anti-mouse IgG (1:500, Invitrogen) secondary antibodies.

### Statistical analysis

Each experiment was repeated at least three times. The values were analyzed using a t-test. p values < 0.05 were considered to be statistically significant.

## Results

### Generation of bovine iPSCs

**Fig 2:**
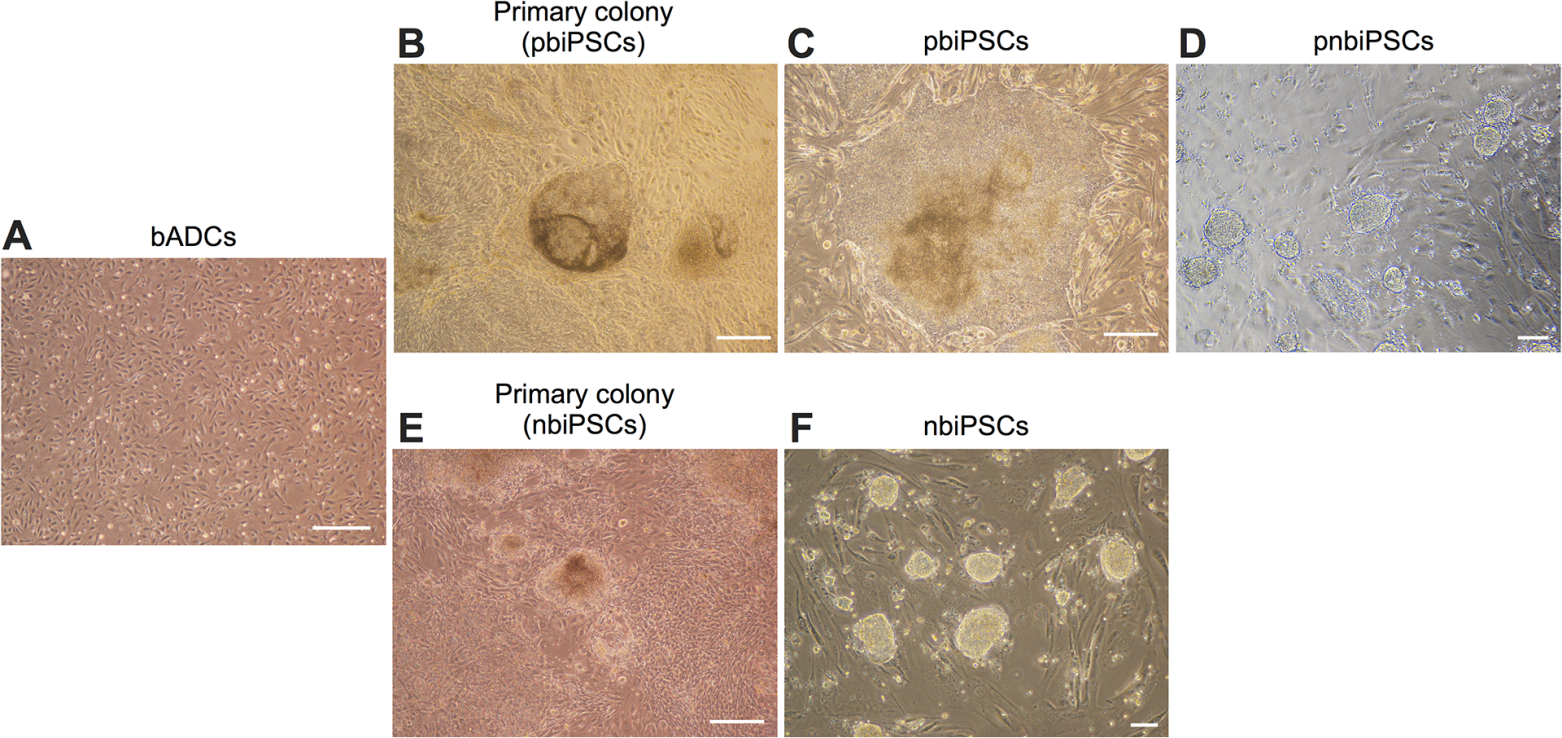
Phase-contrast images of biPSCs established in two different culture conditions. (A) bADCs. (B) Primary colonies appearing in primed cell-culture medium. (C) Established primed-type biPSCs. (D) Colonies converted from the primed to naïve state. (E) Primary colonies appearing in niPSCs medium. (F) Established naïve-type biPSCs. (A)–(C), (E), scale bars = 500 μm. (D), (F), scale bars = 100 μm.

bADCs in culture originally exhibited a heterogeneous population consisting of epithelial and fibroblastic cells (Fig 2A). After transfection with Dox-inducible piggyBac vectors containing reprogramming factors, the bADCS were cultured in piPSC medium, which was similar to medium generally used for maintenance of human ES cells. Around 8 days after Dox addition, primary colonies appeared, and flattened colonies (human ES cell-like) emerged at day 14 (Fig 2B). The efficiency of colony appearance at day 14, relative to the total number of cells reseeded onto the SNL feeder layer at day 5, was 0.01%. These colonies (pbiPSCs) were then picked mechanically and transferred onto a fresh SNL feeder layer. pbiPSCs could be stably propagated and subcultured every 7 days over 70 passages in piPSC medium (Fig 2C).

Addition of 2i and forskolin to culture medium can support naïve characteristics of human iPSCs [9]. In this study, pbiPSCs were dissociated enzymatically and transferred to niPSC medium containing KSR, bLIF, 2i, and forskolin. These cells proliferated and formed mouse ES cell-like colonies with 3-dimensional morphology (Fig 2D). Cells converted from the primed to the naïve state (pnbiPSCs) were maintained in niPSC medium by at least 15 rounds of trypsinization and single-cell dissociation. Furthermore, when the transfected cells were directly cultured in niPSC medium from 8 days after Dox addition, when primary colonies appeared, nbiPSCs colonies with dome-shape and compact morphology emerged at day 14 (Fig 2E). The efficiency of colony appearance at day 14 against the total number of cells, which were reseeded on the SNL feeder layer at day 4, was 0.01%. After passage of nbiPSCs by trypsinization and reseeding onto a fresh SNL feeder layer, cells could be maintained for at least 15 passages by single-cell dissociation every 4 days (Fig 2F). When naïve-type biPSCs (pnbiPSCs and nbiPSCs) were transferred to piPSC medium, they reverted to flattened hESC-like colony morphology, and could be maintained in this medium (retaining the flattened morphology) for over 10 passages.

To test which culture compounds were essential for maintaining naïve characteristics, pnbiPSCs were cultured in different culture conditions, in the presence or absence of GSK3 inhibitor (CH), Mek/Erk inhibitor (PD), and/or forskolin (FK) (S1 Fig). In the absence of all of these compounds (S1A Fig), or in the presence of FK alone (S1D Fig), colonies became more flattened. In the presence of PD, a subset of colonies retained the three-dimensional morphology (S1B Fig), whereas in the presence of CH, most of the colonies had the naïve-type morphology (S1C Fig). Cell number was elevated in the presence of FK (S1I Fig).

### Characterization of primed-type and naïve-type biPSCs

**Fig 3:**
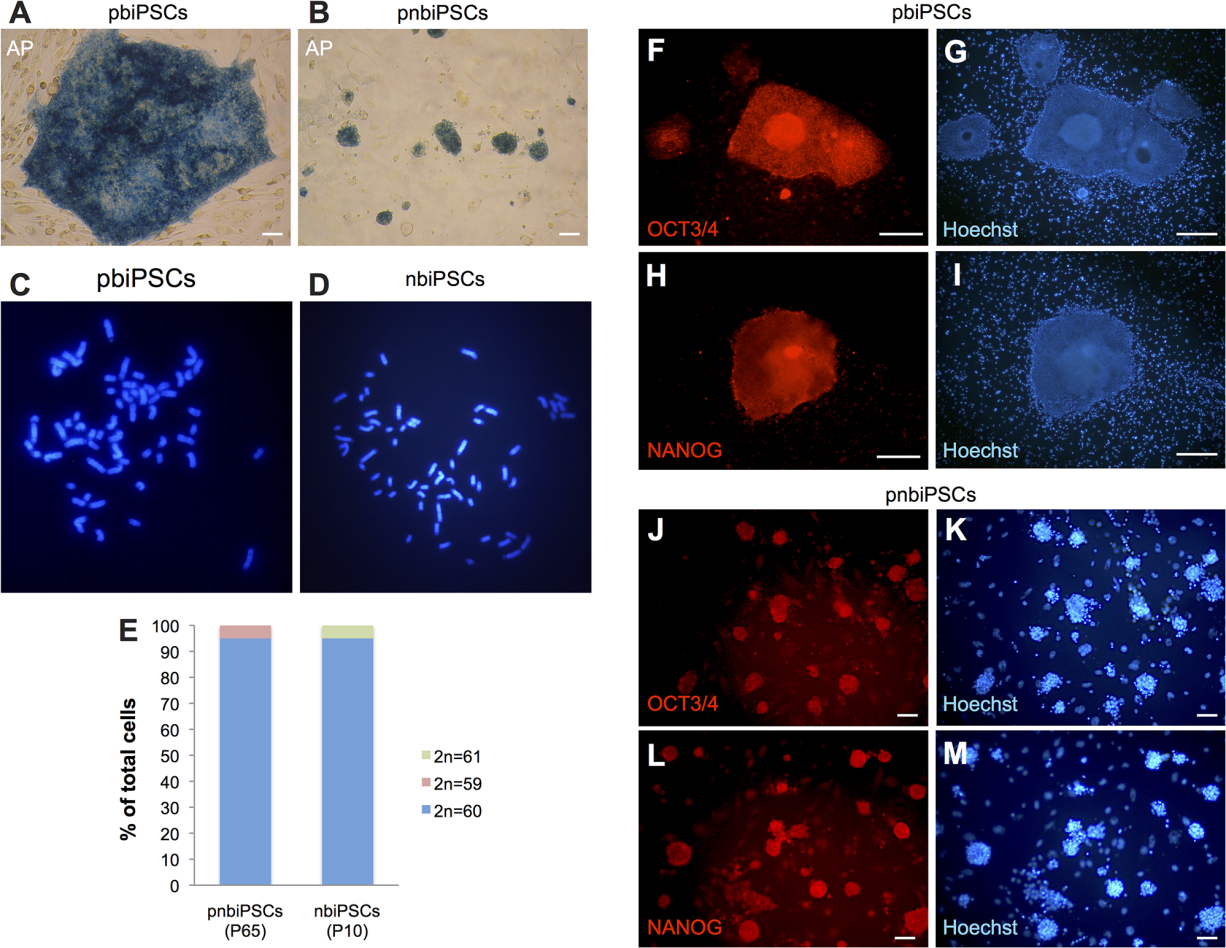
Characterization of biPSCs. (A) Alkaline phosphatase activity in primed-type iPSCs (pbiPSCs). (B) Alkaline phosphatase activity in naïve-type iPSCs derived from pbiPSCs (pnbiPSCs). (C) Karyotyping image of pbiPSCs at passage 65. (D) Karyotyping image of nbiPSCs at passage 10. (E) Proportion of cells with the indicated number of chromosomes (n = 20). (F)–(I) OCT3⁄4 (F, OCT3⁄4 staining; G, Hoechst staining) and NANOG (H, NANOG staining; I, Hoechst staining) expression in pbiPSCs. (J)–(M) OCT3⁄4 (J, OCT3⁄4 staining; K, Hoechst staining) and NANOG (L, NANOG staining; M, Hoechst staining) expression in pnbiPSCs. (A), (B), (J)–(M), scale bars = 100 μm. (F)–(G), scale bars = 500 μm.

Both primed-type and naïve-type biPSCs were positive for alkaline phosphatase (AP) activity (Fig 3A, 3B and S2A, S2H Fig). In the karyotype analysis, 19 out of 20 (95%) pbiPSCs or nbiPSCs with metaphase plates had the normal number (2n = 60) of chromosomes, even after they were propagated over 65 passages (Fig 3C–3E); one pbiPSC had 59 chromosomes, and one nbiPSC had 61 chromosomes.

**Fig 4:**
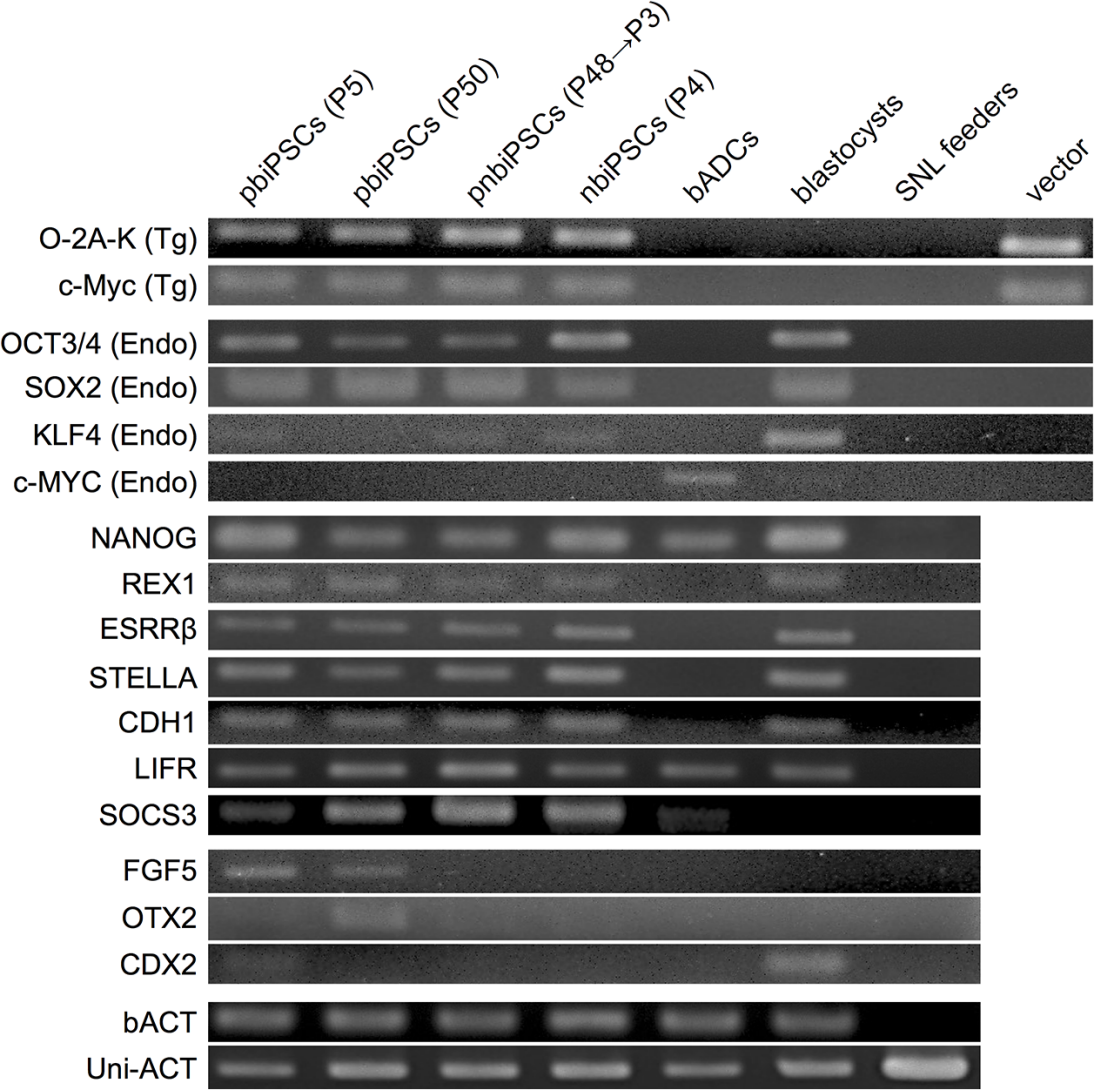
Endogenous and exogenous expression of genes specific to undifferentiated ESCs in biPSCs. mRNA expression was evaluated by reverse-transcription polymerase chain reaction (RT-PCR). pbiPSCs (P5), primed-type iPSCs at passage 5; pbiPSCs (P50), primed-type iPSCs at passage 50; pnbiPSCs, naïve-type iPSCs at passage 3 converted from primed-type iPSCs at passage 48; nbiPSCs, naïve-type iPSCs cultured under naïve medium from primary culture; bADCs, bovine amnion-derived cells; SNL feeder, SNL feeder cells; vector, plasmid DNA of PB vectors; bACT, bovine β-ACTIN specific for cattle; Uni-ACT, universal β-ACTIN that reacts with both cattle and mice.

Immunocytochemistry analysis revealed that biPSCs expressed exogenous and/or endogenous OCT3/4 and NANOG (Fig 3F–3I, 3J–3M, and S2B–S2G, S2I–S2L Fig). RT-PCR analysis also revealed that these cells expressed the pluripotency-related genes including *OCT3/4*, *SOX2*, *NANOG*, *E-CADHERIN* (*CDH1*), as well as naïve mouse iPSC–specific genes such as *REX1*, *ESRRβ*, *STELLA*, *LIFR*, and *SOCS*3 (Fig 4). On the other hand, *FGF5*, and *OTX2*, a marker gene for primed human iPSCs, was only detected in pbiPSCs (Fig 4).

**Fig 5:**
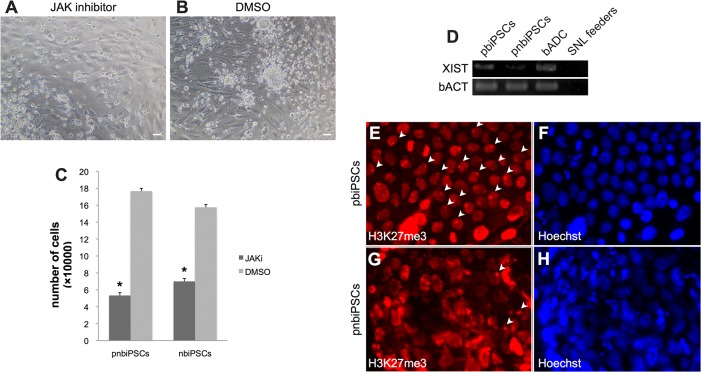
Naïve-type features of iPSCs. (A) pnbiPSCs cultured for 4 days in niPSC medium in the presence of JAK inhibitor. (B) pnbiPSCs cultured in the presence of DMSO. (C) The number of cells cultured in the presence of JAK inhibitor or DMSO (*p < 0.05). (D) *XIST* expression evaluated in pbiPSCs, but not in pnbiPSCs. Immunocytochemistry images of methylation status at H3K27me3 sites (E, pbiPSCs; F, Hoechst staining; G, pnbiPSCs; H, Hoechst staining). Arrowheads indicate puncta of H3K27me3. (A), (B), scale bars = 500 μm.

To investigate the signaling pathways required for the maintenance of self-renewal of biPSCs, naïve-type biPSCs were cultured with JAK inhibitor in the absence of bLIF for 4 days. Under these culture conditions, the number of cells was significantly reduced, by more than 60% in pnbiPSCs (Fig 5A, 5B and 5C) and 50% in nbiPSCs (S2M and S2N Fig and Fig 5C). By contrast, judging by their appearance, the proliferation of pbiPSCs was not affected in this culture condition (S2O and S2P Fig).

Next, we examined X-chromosome inactivation states in the established biPSC cell lines. RT-PCR analysis revealed that *XIST* mRNA was not expressed in pnbiPSCs, but it was expressed in pbiPSCs and female bADCs (used as a positive control) (Fig 5D). On the other hand, histone H3K27 trimethylation signals associated with X chromosomes inactivation were observed in pbiPSCs, but not in pnbiPSCs (Fig 5E–5H).

When biPSCs were cultured in the absence of Dox, they readily changed their morphology and differentiated (S3A and S3B Fig). RT-PCR analysis revealed that in the absence of Dox, pbiPSCs and pnbiPSCs no longer expressed exogenous transgenes and endogenous pluripotent genes, such as *OCT3/4*, *ESRRβ*, and *STELLA*, but instead began to express endogenous *c-MYC* (S3C Fig).

### Differentiation potential of biPSCs

**Fig 6:**
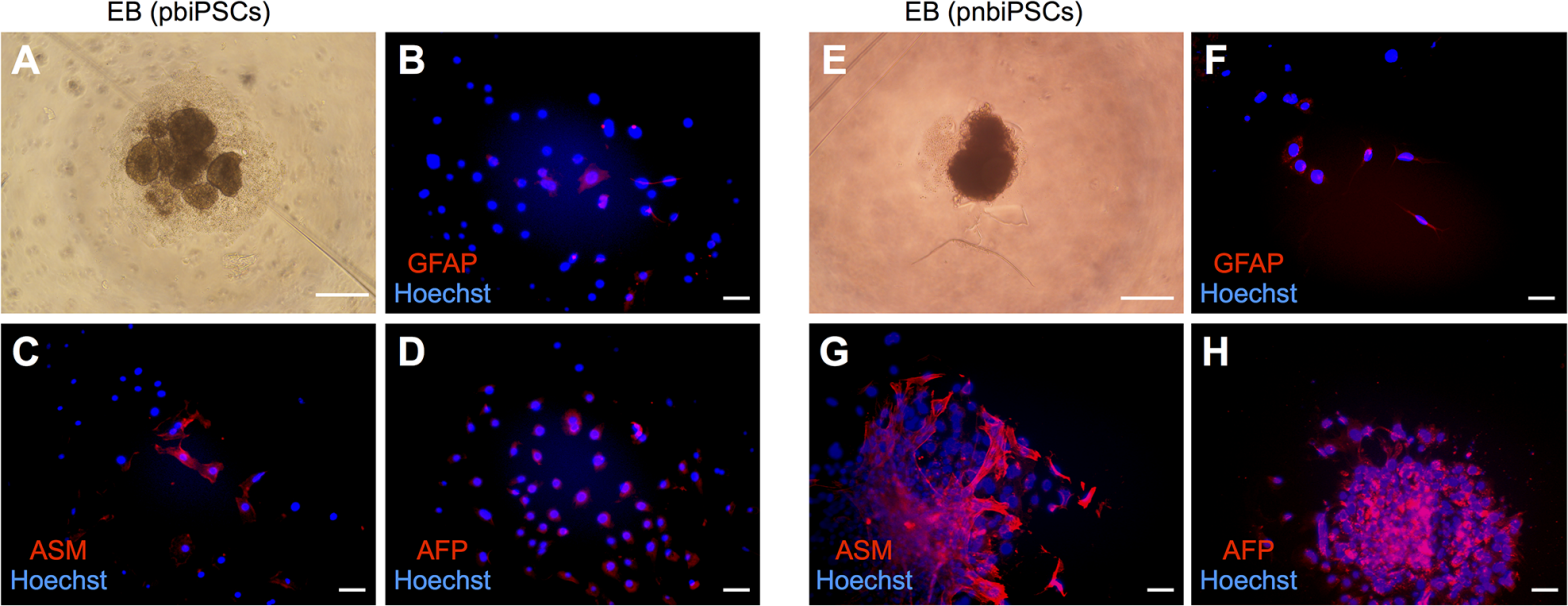
Differentiation potential of biPSCs in culture. (A) Embryoid body formation of pbiPSCs grown for 6 days in low cell-adhesion dishes. Immunocytochemical staining for markers for the three germ-layer in differentiated cells derived from pbiPSCs. α-fetoprotein (B, endoderm), actin smooth muscle (C, mesoderm), and glial fibrillary acidic protein (D, ectoderm) were used as markers. (E) Embryoid body formation by pnbiPSCs. Immunocytochemical staining for α-fetoprotein (F), actin smooth muscle (G), glial fibrillary acidic protein (H). (A), (E), scale bars = 500 μm. (B)–(D), (F)–(H), scale bars = 100 μm.

When biPSCs were induced to differentiate in low-adhesion culture dishes for 6 days, they formed embryoid bodies (Fig 6A, 6E and S4A, S4B and S4M Fig). After they were cultured for another 6 days, they differentiated into all three germ layers, as assessed by immunocytochemistry using specific markers for each layer: GFAP for the ectoderm, ASM for the mesoderm, and AFP for the endoderm (Fig 6B–6D, 6F–6H and S4C–S4K, S4N–S4S Fig). We also assessed the *in vitro* differentiation potential of pnbiPSCs by RT-PCR using specific markers such as *VIMENTIN* (*VIM*) for ectoderm, *BMP4* for mesoderm, and AFP for endoderm (S4L Fig).

**Fig 7:**
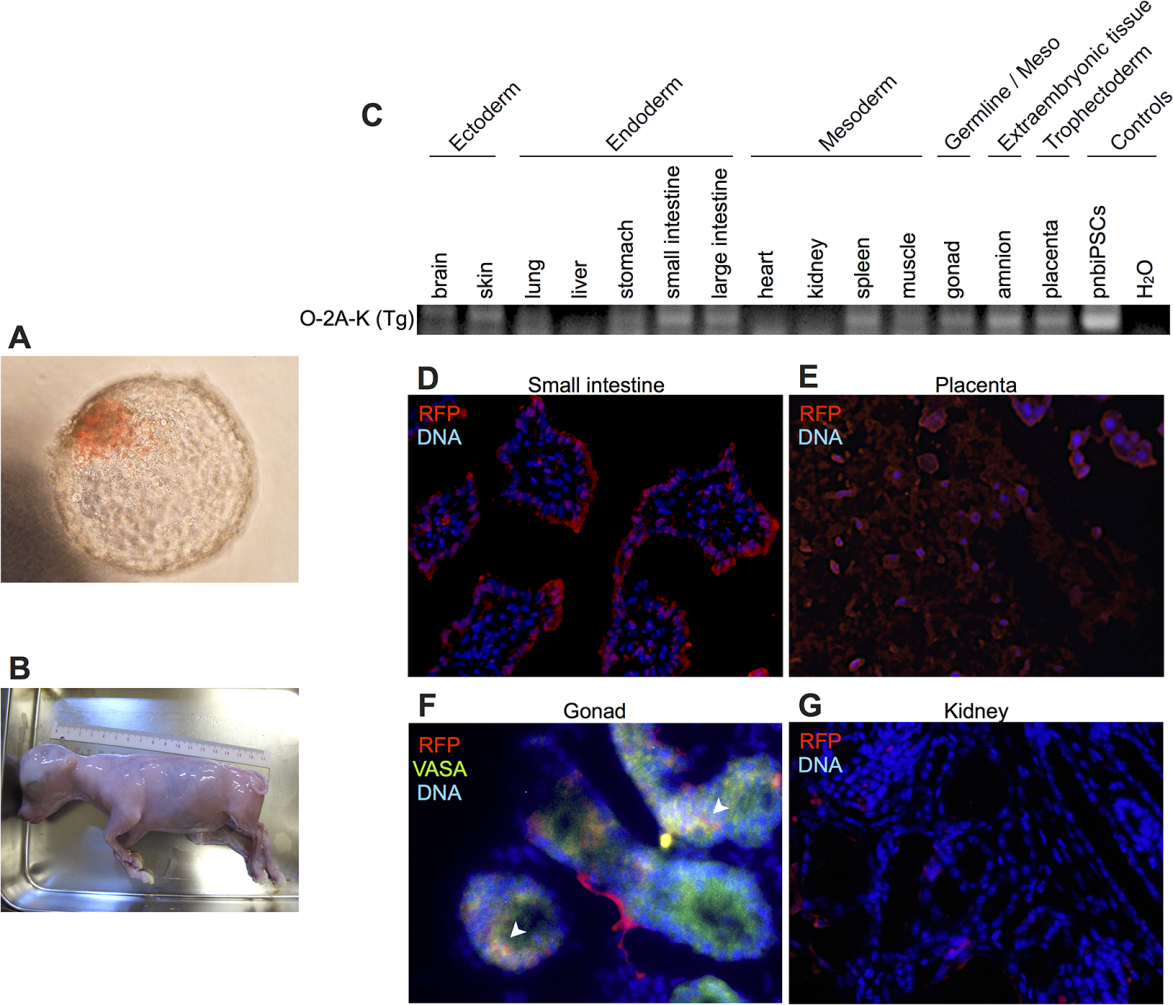
Production of chimeric fetuses from bovine embryos using the aggregation method. (A) Naïve-type biPSCs expressing Tag-RFP were aggregated with host at the 8- to 16-cell stage of *in vitro* fertilized embryos. (B) Chimeric fetuses at day 90 of gestation derived from aggregated embryos. (C) PCR analysis using transgene-specific primers for genomically integrated Oct3/4-2A-Klf4 sequences in 14 tissues. Genomic DNA isolated from pnbiPSCs was used as a positive control. H_2_O was used as a negative control (buffer alone for RT-PCR). Immunofluorescence analysis showing the distribution of pnbiPSC-derived cells (RFP-positive with red signals) in the small intestine (D), placenta (E), gonad (F, VASA-positive cells with green signals; arrowheads indicate the portion that is double-positive for RFP and VASA), and kidney (G) of the chimeric fetus. Nuclei were stained with DAPI (blue).

To test whether naïve-type biPSCs had the potential to associate with the normal development of bovine embryos, we generated naïve-type biPSCs expressing Tag-RFP and aggregated them with 8- to 16-cell stage embryos. In preliminary experiments, pbiPSCs were allowed to aggregate with 8- to 16-cell stage embryos by two different ways of cell dissociation. When we trypsinized pbiPSCs and aggregated them with embryos, the cells were scattered around and didn’t aggregate with embryonic cells. On the other hand, when we mechanically split pbiPSCs and aggregated with embryos, cell clumps were formed outside embryos, however, didn’t make aggregation. Meanwhile, naïve-type biPSCs developed normally into blastocysts, to the same degree as aggregation performed only with 8- to 16-cell stage embryos (Table 1). Judging from RFP fluorescence, pnbiPSCs and nbiPSCs were successfully incorporated into the ICM region of blastocyst stage embryos (Fig 7A). In addition, we sometimes observed incorporation into both the ICM and TE regions (13.9% and 34.4% in the case of pnbiPSCs and nbiPSCs, respectively; Table 1). By contrast, we did not observe incorporation of bADCs expressing RFP into the embryos (Table 1).

**Table 1:** Aggregation of pnbiPSCs into *in vitro* fertilized embryos, and their development *in vitro*.

			Chimeric blastocysts (%)		
Donor cells	No. of aggregated embryos	No. of blastcysts developed (%)	No. contributed to ICM	No. contributed to both ICM and TE	No. contributed to TE
pnbiPSCs	60	38 (63.3)	31 (86.1)	5 (13.9)	0 (0)
nbiPSCs	60	35 (58.3)	21 (65.6)	11 (34.4)	0 (0)
bADCs*	60	35 (58.3)	0 (0)	0 (0)	0 (0)
Embryo only	60	34 (56.7)	0 (0)	0 (0)	0 (0)

*RFP vector was introduced

Chimeric embryos formed by aggregation of 8- to 16-cell bovine embryos and pnbiPSCs were subsequently transferred to the uteruses of a surrogate mother in order to trace their developmental capacity *in vivo*. Three chimeric embryos were transferred into each uterine horn, and the resultant fetuses were recovered at 90 days after transplantation (Fig 7B). All of the fetuses developed normally. The fetuses were then isolated and separated by individual organs/tissues representing ectoderm (brain, skin), endoderm (lung, liver, stomach, small intestine, large intestine), mesoderm (heart, kidney, spleen, muscle), germline (gonad), fetal membrane (amnion) and trophectoderm (placenta). PCR analysis of integrated Oct3/4-2A-Klf4 sequences were performed in each fetal tissue. Eleven out of 14 tissues (brain, skin, lung, stomach, small intestine, large intestine, spleen, muscle, gonad, amnion, and placenta) showed evidence of a pnbiPSC contribution (Fig 7C).

PCR analysis revealed the contribution of pnbiPSCs to chimeric fetuses. In addition, we performed immunofluorescence analysis on frozen tissue sections of small intestine, kidney, placenta, and gonad. Red fluorescence from TagRFP-expressing vector was observed in the small intestine, placenta, and gonad (Fig 7D, 7E and 7F). By contrast, fluorescence was faint or absent in the kidney (Fig 7G). These immunofluorescence results were coincident with those of PCR analysis. Moreover, in the gonad, some of the cells were double-positive for RFP (red) and VASA (green), a primordial germ cell marker (Fig 7F, arrowheads).

## Discussion

Since the first generation of iPSCs in mouse [1], a large number of studies of iPSCs have been performed in both mouse and human. Despite numerous attempts, however, very few studies have reported the generation of iPSCs from somatic cells or ESCs from preimplantation embryos in other species. In cattle, Cao et al. [33], Ozawa et al. [34], and Furusawa et al. [35] reported the generation of bovine embryonic stem cell–like cells, and Sumer et al [17] reported the generation of primed biPSCs, but also described the difficulty of derivation and maintenance of these cells. Most iPSCs reported in non-rodent species were primed iPSCs [10–17], which have a limited capacity to produce chimeras relative to naïve iPSCs [3, 4, 8]. Here, we report the generation of two different types (i.e., primed and naïve) of biPSCs established from bADCs by introducing Dox-inducible PB vectors. Both types had characteristics of pluripotent stem cells; in particular, the naïve-type biPSCs exhibited several characteristics of pluripotent cells, comparable to those of naïve mouse iPSCs, and contributed to the ICM of bovine host blastocysts and chimeric fetuses.

Sumer et al. [17] reported previously that generation of biPSCs by retroviral delivery and the ectopic expression of *POU5F1*, *SOX2*, *KLF4*, *c-MYC*, and *NANOG*, and demonstrated that ectopic expression of *NANOG* is necessary for the generation and maintenance of biPSCs from bovine fetal fibroblasts. In this study, we were able to generate biPSCs without ectopic expression of *NANOG*, possibly because the bADCs used in this study expressed intrinsic endogenous *NANOG* (Fig 4). Therefore, ectopic expression of *NANOG* may not be necessary for production and maintenance of biPSCs. In addition, studies on human and mouse iPSCs have suggested that reprogramming via introduction of transcription factors in ADCs is more efficient and faster than in fibroblasts [24–26] probably because ADCs in mouse and human express high endogenous levels of *Klf4*, *c-Myc*, and *Ronin*, which support proliferation and self-renewal of iPSCs [25]. When we used bovine embryonic fibroblast cells and transfected them with the same combination of transcription factors, iPSC-like colonies appeared, but no stable iPSC lines were established (data not shown). Moreover, when we cultured bADCs in low-adhesion culture dishes for 6 days, they formed EB-like cell masses and differentiated a part of cell types representing each germ layer after culturing on gelatin-coated dishes for another 6 days. A previous report [36] showed that a subpopulation of human ADCs exhibited stem cell-like characteristics and an ability to differentiate into various cell types in tridermic layer. In our study, bADCs also exhibited the expression of *NANOG* as well as several lineage specification markers such as *VIM* (ectoderm marker) and *BMP4* (mesoderm marker). Thus, bADCs have stem cell-like characteristics and represent an appropriate cell source for generation of iPSCs in cattle.

The choice of reprogramming components, and the order of these factors in the vector, also affect the efficiency of reprogramming of somatic cells and the generation of iPSCs. Okita et al. [31] reported that expression of Oct3/4, Klf4, and then Sox2 (OKS) in that order in polycistronic vectors improves efficiency of reprogramming and facilitates the generation of murine iPSCs. Tsukiyama et al. [18] reported that cell reprogramming induced by the combination of OKS and c-Myc (M) vectors (OKS + M) was more efficient than that induced by polycistronic MKOS vectors. When we used MKOS vectors instead of OKS + M vectors, no iPSC-like colonies appeared (data not shown), whereas OKS + M vectors could generate iPSC-like colonies with an efficiency of 0.01%. Therefore, the OKS + M vector combination is also useful for generation of iPSCs in cattle.

The biPSCs reported by Sumer et al. [17] appeared in the primed state, as judged by their morphology and the use of a culture medium containing FGF. In this study, when the primed-type biPSCs were passaged and maintained in the niPSCs medium containing bLIF, 2i, and forskolin, naïve-type biPSCs appeared. These cells exhibited several hallmarks of naïve PSCs, such as the expression of naïve marker genes (including the STAT3 target *SOCS3* [37]) LIF-dependent proliferation [3, 4], and reactivation of the X chromosome (X_a_X_a_) [3, 4, 9]. In humans, primed iPSCs can be converted into naïve iPSCs by the addition of 2i and forskolin to the culture medium [9]. In our experiment, once naïve-type cells appeared, only the addition of bLIF + GSK3β inhibitor could maintain them in this state (S1C Fig), indicating that the continuous expression of transcription factors in naïve-type cells could substitute for the effects of Mek/Erk inhibitor and forskolin. On the other hand, our primed-type biPSCs exhibited a flattened morphology, LIF-independent proliferation, inactivation of the X chromosome (X_a_X_i_), and the expression of primed marker genes such as *FGF5* (Fig 4); however, they also expressed naïve marker genes such as *REX1*, *ESRRβ*, *STELLA*, *LIFR*, and *SOCS3* (Fig 4). Recent reports describe intermediate states of cells of murine ESCs or iPSCs that share characteristics with the primed- and naïve-cell states [38, 39], even in primed culture conditions [39]. These intermediate cells express both primed marker genes, such as *Fgf5*, and naïve marker genes such as *Rex1*, *Esrrβ*, and *Stella*. Tsukiyama et al. [38] also showed that intermediate cells do not depend on the LIF-Jak/Stat pathway for proliferation. These data are in agreement with our findings; hence, our primed-type biPSCs should be considered as similar to intermediate cells.

In the absence of Dox, biPSCs readily changed their morphology and no longer expressed exogenous transgenes and endogenous pluripotent genes. Instead, they began to express endogenous *c-MYC* (S3C Fig). These results indicated that transgene expression could be controlled by the removal of Dox, and that continuous transgene expression is necessary to maintain biPSCs in a pluripotent state. Optimal culture conditions for the establishment and maintenance of ESCs vary among species [40–42]. Most of the iPSC lines established in non-rodent species depend on continuous transgene expression to maintain their pluripotency [12–14, 17, 43]. More recently, naïve human ESCs or iPSCs can be established without transgene expression in the presence of LIF, FGF2, and TGFβ1 (a member of the TGF-β superfamily, and the same as activin A) and inhibitors against four signaling pathways (ERK1/2, GSK3β, JNK, and p38) for stable cell propagation [44]. Therefore, further studies are required in order to determine the optimal culture conditions for maintaining established biPSCs in the absence of Dox. This study provides a model for generating authentic naïve-type biPSCs under the control of transgenes.

The biPSCs we established exhibited many features of pluripotency; however, they did not form teratomas in nude mice (BALB⁄c nu/nu, data not shown). The difficulty of developing mature teratomas from naïve-like human iPSCs has been described previously [6, 45, 46], and the specific strain of immunodeficient mouse affects teratoma formation [47]. On the other hand, biPSCs could form embryoid bodies and differentiate into all three germ layers *in vitro*. In addition, they were incorporated into ICM, and sometimes into TE, after aggregation with 8- to 16-cell stage embryos. These observations led us to examine the potential of biPSCs to contribute to chimeric fetuses. Although primed-type biPSCs could propagate stably for more than 70 passages, naïve-type biPSCs were somewhat difficult to maintain, and could be propagated for only 10 to 15 passages; therefore, pnbiPSCs (derived from pbiPSCs) were employed for aggregation and transplantation. Aggregated embryos transplanted into the uteruses of surrogate mothers successfully produced three chimeric fetuses. Although the contribution of pnbiPSCs to some tissues was faint or absent, these cells had the potential to differentiate into all three germ layers, trophectoderm, and potentially germline *in vivo*. Incorporation into the trophectoderm has not been observed in mice, but has been frequently observed in pigs [48]. This difference is possibly due to interspecies diversity in *OCT3/4* regulation, resulting in changes in Cdx2:Oct4 ratios and the relative timing of trophectoderm commitment observed not only in cattle but also in human, pig, and rabbit blastocysts [49]. Our study provides the first demonstration that biPSCs can contribute to chimeric fetuses and differentiate into all tissues, including extraembryonic tissues. The next step to be achieved is the production of adult chimeras and offspring.

In summary, we generated two different types of biPSCs from bADCs using Dox-inducible PB vectors. Our results show for the first time that biPSCs meet the criteria for naïve iPSCs and have the capacity to contribute to chimeric fetuses, including the germ cell lineage. These cell lines should facilitate the optimization of culture conditions for generation and and maintenance of bovine iPSCs that have the potential to generate chimeric offspring.

## Supporting Information

S1 Fig Appearance of naïve-type biPSCs cultured under different conditions. pnbiPSCs cultured only with bLIF (A), bLIF+CH (B), bLIF+PD (C), bLIF+FK (D), LIF+CH+PD (E), bLIF+PD+FK (F), bLIF+FK+CH (G), and bLIF+FK+CH+FK (H). (I) Numbers of growing cells after cultivation in different conditions for 4 days. (A)–(H), scale bars = 500 μm.
(TIFF)

S2 Fig Characterization of biPSCs. (A) Alkaline phosphatase activity in nbiPSCs. OCT3⁄4 (B, RFP-positive image; C, OCT3⁄4 staining; D, Hoechst staining) and NANOG (E, RFP-positive image; F, NANOG staining; G, Hoechst staining) expression in nbiPSCs expressing RFP. (H) Alkaline phosphatase activity in bADCs. OCT3⁄4 (I, OCT3⁄4 staining; J, Hoechst staining) and NANOG (K, NANOG staining; L, Hoechst staining) expression in bADCs. (M) nbiPSCs cultured in the presence of JAK inhibitor for 4 days. (N) nbiPSCs cultured in the presence of DMSO. (O) pbiPSCs cultured in the presence of JAK inhibitor for 4 days. (P) pbiPSCs cultured in the presence of DMSO. (A)–(L) scale bars = 100 μm. (M)–(P) scale bars = 500 μm.
(TIF)

S3 Fig biPSCs cultured in the absence of Dox (- Dox). (A) Phase-contrast image of pnbiPSCs cultured in the absence of Dox for 4 days. (B) Phase-contrast image of pbiPSCs cultured in the absence of Dox for 7 days. (C) Endogenous and exogenous gene expression in biPSCs cultured in the absence of Dox. (A), (B), scale bars = 500 μm.
(TIFF)

S4 Fig Differentiation potential of nbiPSCs expressing RFP. (A) Embryoid body formation of nbiPSCs grown in low cell-adhesion dishes for 6 days. Glial fibrillary acidic protein (C, RFP-positive image; D, glial fibrillary acidic protein; E, Hoechst staining), actin smooth muscle (F, RFP-positive image; G, actin smooth muscle; H, Hoechst staining) and α-fetoprotein (I, RFP-positive image; J, α-fetoprotein; K, Hoechst staining) were used for the markers. (L) Gene-expression profile of pnbiPSCs after embryoid body differentiation. EB, Embryoid body; EB dif, EB-derived cells cultured for an additional 6 days on a gelatin-coated dish; VIM, VIMENTIN. (M) Embryoid body formation of bADCs grown in low cell-adhesion dishes for 6 days. Glial fibrillary acidic protein (N, glial fibrillary acidic protein; O, Hoechst staining), actin smooth muscle (P, actin smooth muscle; Q, Hoechst staining) and α-fetoprotein (R, α-fetoprotein; S, Hoechst staining) were used for the markers. (A), (B), (M) scale bars = 500 μm. (C)–(S) scale bars = 70 μm.
(TIF)

S1 Table Primer sequences. (XLSX)

## References

[1] Takahashi K, Yamanaka S. Induction of pluripotent stem cells from mouse embryonic and adult fibroblast cultures by defined factors. Cell. 2006;126(4):663–76. Epub 2006/08/15. 10.1016/j.cell.2006.07.024 . 16904174

[2] Takahashi K, Tanabe K, Ohnuki M, Narita M, Ichisaka T, Tomoda K, et al Induction of pluripotent stem cells from adult human fibroblasts by defined factors. Cell. 2007;131(5):861–72. Epub 2007/11/24. 10.1016/j.cell.2007.11.019 . 18035408

[3] Nichols J, Smith A. Naive and primed pluripotent states. Cell Stem Cell. 2009;4(6):487–92. 10.1016/j.stem.2009.05.015 . 19497275

[4] Hanna JH, Saha K, Jaenisch R. Pluripotency and cellular reprogramming: facts, hypotheses, unresolved issues. Cell. 2010;143(4):508–25. Epub 2010/11/16. 10.1016/j.cell.2010.10.008 ; PubMed Central PMCID: PMCPmc3032267. 21074044 PMC3032267

[5] Kinoshita M. How are pluripotent cells captured in culture? Reproductive Medicine and Biology. 2014:1–14. 10.1007/s12522-014-0199-8 PMC4490168 26161037

[6] Buecker C, Chen HH, Polo JM, Daheron L, Bu L, Barakat TS, et al A murine ESC-like state facilitates transgenesis and homologous recombination in human pluripotent stem cells. Cell Stem Cell. 2010;6(6):535–46. 10.1016/j.stem.2010.05.003 20569691 PMC3162213

[7] Silva J, Nichols J, Theunissen TW, Guo G, van Oosten AL, Barrandon O, et al Nanog is the gateway to the pluripotent ground state. Cell. 2009;138(4):722–37. 10.1016/j.cell.2009.07.039 19703398 PMC3437554

[8] Tesar PJ, Chenoweth JG, Brook FA, Davies TJ, Evans EP, Mack DL, et al New cell lines from mouse epiblast share defining features with human embryonic stem cells. Nature. 2007;448(7150):196–9. 10.1038/nature05972 . 17597760

[9] Hanna J, Cheng AW, Saha K, Kim J, Lengner CJ, Soldner F, et al Human embryonic stem cells with biological and epigenetic characteristics similar to those of mouse ESCs. Proc Natl Acad Sci U S A. 2010;107(20):9222–7. 10.1073/pnas.1004584107 20442331 PMC2889088

[10] Liu H, Zhu F, Yong J, Zhang P, Hou P, Li H, et al Generation of induced pluripotent stem cells from adult rhesus monkey fibroblasts. Cell Stem Cell. 2008;3(6):587–90. 10.1016/j.stem.2008.10.014 . 19041774

[11] Tomioka I, Maeda T, Shimada H, Kawai K, Okada Y, Igarashi H, et al Generating induced pluripotent stem cells from common marmoset (Callithrix jacchus) fetal liver cells using defined factors, including Lin28. Genes Cells. 2010;15(9):959–69. 10.1111/j.1365-2443.2010.01437.x 20670273 PMC2970909

[12] Wu Z, Chen J, Ren J, Bao L, Liao J, Cui C, et al Generation of pig induced pluripotent stem cells with a drug-inducible system. J Mol Cell Biol. 2009;1(1):46–54. 10.1093/jmcb/mjp003 . 19502222

[13] Ezashi T, Telugu BP, Alexenko AP, Sachdev S, Sinha S, Roberts RM. Derivation of induced pluripotent stem cells from pig somatic cells. Proc Natl Acad Sci U S A. 2009;106(27):10993–8. 10.1073/pnas.0905284106 19541600 PMC2698893

[14] Esteban MA, Xu J, Yang J, Peng M, Qin D, Li W, et al Generation of induced pluripotent stem cell lines from Tibetan miniature pig. J Biol Chem. 2009;284(26):17634–40. 10.1074/jbc.M109.008938 19376775 PMC2719402

[15] Montserrat N, Bahima EG, Batlle L, Häfner S, Rodrigues AM, González F, et al Generation of pig iPS cells: a model for cell therapy. J Cardiovasc Transl Res. 2011;4(2):121–30. 10.1007/s12265-010-9233-3 . 21088946

[16] Honda A, Hirose M, Hatori M, Matoba S, Miyoshi H, Inoue K, et al Generation of induced pluripotent stem cells in rabbits: potential experimental models for human regenerative medicine. J Biol Chem. 2010;285(41):31362–9. 10.1074/jbc.M110.150540 20670936 PMC2951210

[17] Sumer H, Liu J, Malaver-Ortega LF, Lim ML, Khodadadi K, Verma PJ. NANOG is a key factor for induction of pluripotency in bovine adult fibroblasts. J Anim Sci. 2011;89(9):2708–16. 10.2527/jas.2010-3666 . 21478453

[18] Tsukiyama T, Asano R, Kawaguchi T, Kim N, Yamada M, Minami N, et al Simple and efficient method for generation of induced pluripotent stem cells using piggyBac transposition of doxycycline-inducible factors and an EOS reporter system. Genes Cells. 2011;16(7):815–25. Epub 2011/06/11. 10.1111/j.1365-2443.2011.01528.x . 21658168

[19] Kaji K, Norrby K, Paca A, Mileikovsky M, Mohseni P, Woltjen K. Virus-free induction of pluripotency and subsequent excision of reprogramming factors. Nature. 2009;458(7239):771–5. 10.1038/nature07864 19252477 PMC2667910

[20] Woltjen K, Michael IP, Mohseni P, Desai R, Mileikovsky M, Hämäläinen R, et al piggyBac transposition reprograms fibroblasts to induced pluripotent stem cells. Nature. 2009;458(7239):766–70. 10.1038/nature07863 19252478 PMC3758996

[21] Yusa K, Rad R, Takeda J, Bradley A. Generation of transgene-free induced pluripotent mouse stem cells by the piggyBac transposon. Nat Methods. 2009;6(5):363–9. 10.1038/nmeth.1323 19337237 PMC2677165

[22] Aasen T, Raya A, Barrero MJ, Garreta E, Consiglio A, Gonzalez F, et al Efficient and rapid generation of induced pluripotent stem cells from human keratinocytes. Nat Biotechnol. 2008;26(11):1276–84. 10.1038/nbt.1503 . 18931654

[23] Kim JB, Zaehres H, Wu G, Gentile L, Ko K, Sebastiano V, et al Pluripotent stem cells induced from adult neural stem cells by reprogramming with two factors. Nature. 2008;454(7204):646–50. 10.1038/nature07061 . 18594515

[24] Li C, Zhou J, Shi G, Ma Y, Yang Y, Gu J, et al Pluripotency can be rapidly and efficiently induced in human amniotic fluid-derived cells. Hum Mol Genet. 2009;18(22):4340–9. 10.1093/hmg/ddp386 . 19679563

[25] Nagata S, Toyoda M, Yamaguchi S, Hirano K, Makino H, Nishino K, et al Efficient reprogramming of human and mouse primary extra-embryonic cells to pluripotent stem cells. Genes Cells. 2009;14(12):1395–404. 10.1111/j.1365-2443.2009.01356.x . 19912344

[26] Zhao HX, Li Y, Jin HF, Xie L, Liu C, Jiang F, et al Rapid and efficient reprogramming of human amnion-derived cells into pluripotency by three factors OCT4/SOX2/NANOG. Differentiation. 2010;80(2–3):123–9. 10.1016/j.diff.2010.03.002 . 20510497

[27] Niwa H, Yamamura K, Miyazaki J. Efficient selection for high-expression transfectants with a novel eukaryotic vector. Gene. 1991;108(2):193–9. . 1660837 10.1016/0378-1119(91)90434-d

[28] McMahon AP, Bradley A. The Wnt-1 (int-1) proto-oncogene is required for development of a large region of the mouse brain. Cell. 1990;62(6):1073–85. . 2205396 10.1016/0092-8674(90)90385-r

[29] Okita K, Ichisaka T, Yamanaka S. Generation of germline-competent induced pluripotent stem cells. Nature. 2007;448(7151):313–7. 10.1038/nature05934 . 17554338

[30] Yusa K, Zhou L, Li MA, Bradley A, Craig NL. A hyperactive piggyBac transposase for mammalian applications. Proc Natl Acad Sci U S A. 2011;108(4):1531–6. 10.1073/pnas.1008322108 21205896 PMC3029773

[31] Okita K, Nakagawa M, Hyenjong H, Ichisaka T, Yamanaka S. Generation of mouse induced pluripotent stem cells without viral vectors. Science. 2008;322(5903):949–53. 10.1126/science.1164270 . 18845712

[32] Booth PJ, Tan SJ, Reipurth R, Holm P, Callesen H. Simplification of bovine somatic cell nuclear transfer by application of a zona-free manipulation technique. Cloning Stem Cells. 2001;3(3):139–50. 10.1089/153623001753205098 . 11945223

[33] Cao S, Wang F, Chen Z, Liu Z, Mei C, Wu H, et al Isolation and culture of primary bovine embryonic stem cell colonies by a novel method. J Exp Zool A Ecol Genet Physiol. 2009;311(5):368–76. 10.1002/jez.535 . 19340839

[34] Ozawa M, Sakatani M, Hankowski KE, Terada N, Dobbs KB, Hansen PJ. Importance of culture conditions during the morula-to-blastocyst period on capacity of inner cell-mass cells of bovine blastocysts for establishment of self-renewing pluripotent cells. Theriogenology. 2012;78(6):1243–51.e1-2. 10.1016/j.theriogenology.2012.05.020 . 22898023

[35] Furusawa T, Ohkoshi K, Kimura K, Matsuyama S, Akagi S, Kaneda M, et al Characteristics of bovine inner cell mass-derived cell lines and their fate in chimeric conceptuses. Biol Reprod. 2013;89(2):28 10.1095/biolreprod.112.106641 . 23782837

[36] Miki T, Lehmann T, Cai H, Stolz DB, Strom SC. Stem cell characteristics of amniotic epithelial cells. Stem Cells. 2005;23(10):1549–59. 10.1634/stemcells.2004-0357 . 16081662

[37] van Oosten AL, Costa Y, Smith A, Silva JC. JAK/STAT3 signalling is sufficient and dominant over antagonistic cues for the establishment of naive pluripotency. Nat Commun. 2012;3:817 10.1038/ncomms1822 22569365 PMC3567838

[38] Tsukiyama T, Ohinata Y. A modified EpiSC culture condition containing a GSK3 inhibitor can support germline-competent pluripotency in mice. PLoS One. 2014;9(4):e95329 10.1371/journal.pone.0095329 24736627 PMC3988182

[39] Ozawa M, Kawakami E, Sakamoto R, Shibasaki T, Goto A, Yoshida N. Development of FGF2-dependent pluripotent stem cells showing naive state characteristics from murine preimplantation inner cell mass. Stem Cell Res. 2014;13(1):75–87. 10.1016/j.scr.2014.04.012 . 24835670

[40] Pease S, Braghetta P, Gearing D, Grail D, Williams RL. Isolation of embryonic stem (ES) cells in media supplemented with recombinant leukemia inhibitory factor (LIF). Dev Biol. 1990;141(2):344–52. . 2120094 10.1016/0012-1606(90)90390-5

[41] Xu RH, Peck RM, Li DS, Feng X, Ludwig T, Thomson JA. Basic FGF and suppression of BMP signaling sustain undifferentiated proliferation of human ES cells. Nat Methods. 2005;2(3):185–90. 10.1038/nmeth744 . 15782187

[42] Suemori H, Nakatsuji N. Generation and characterization of monkey embryonic stem cells. Methods Mol Biol. 2006;329:81–9. 10.1385/1-59745-037-5:81 . 16845985

[43] Shimada H, Nakada A, Hashimoto Y, Shigeno K, Shionoya Y, Nakamura T. Generation of canine induced pluripotent stem cells by retroviral transduction and chemical inhibitors. Mol Reprod Dev. 2010;77(1):2 10.1002/mrd.21117 . 19890968

[44] Gafni O, Weinberger L, Mansour AA, Manor YS, Chomsky E, Ben-Yosef D, et al Derivation of novel human ground state naive pluripotent stem cells. Nature. 2013;504(7479):282–6. 10.1038/nature12745 . 24172903

[45] Hirano K, Nagata S, Yamaguchi S, Nakagawa M, Okita K, Kotera H, et al Human and mouse induced pluripotent stem cells are differentially reprogrammed in response to kinase inhibitors. Stem Cells Dev. 2012;21(8):1287–98. 10.1089/scd.2011.0283 . 21882976

[46] Nishishita N, Shikamura M, Takenaka C, Takada N, Fusaki N, Fusak N, et al Generation of virus-free induced pluripotent stem cell clones on a synthetic matrix via a single cell subcloning in the naïve state. PLoS One. 2012;7(6):e38389 10.1371/journal.pone.0038389 22719883 PMC3374798

[47] Drukker M, Katchman H, Katz G, Even-Tov Friedman S, Shezen E, Hornstein E, et al Human embryonic stem cells and their differentiated derivatives are less susceptible to immune rejection than adult cells. Stem Cells. 2006;24(2):221–9. 10.1634/stemcells.2005-0188 . 16109762

[48] Fujishiro SH, Nakano K, Mizukami Y, Azami T, Arai Y, Matsunari H, et al Generation of naive-like porcine-induced pluripotent stem cells capable of contributing to embryonic and fetal development. Stem Cells Dev. 2013;22(3):473–82. 10.1089/scd.2012.0173 22889279 PMC3549629

[49] Berg DK, Smith CS, Pearton DJ, Wells DN, Broadhurst R, Donnison M, et al Trophectoderm lineage determination in cattle. Dev Cell. 2011;20(2):244–55. 10.1016/j.devcel.2011.01.003 . 21316591

