# Supplementary figures and images for: Generation of Naïve Bovine Induced Pluripotent Stem Cells Using PiggyBac Transposition of Doxycycline-Inducible Transcription Factors
Source: PLoS One. 2015 Aug 19;10(8):e0135403. doi: 10.1371/journal.pone.0135403 (PMC4544884; doi:10.1371/journal.pone.0135403)

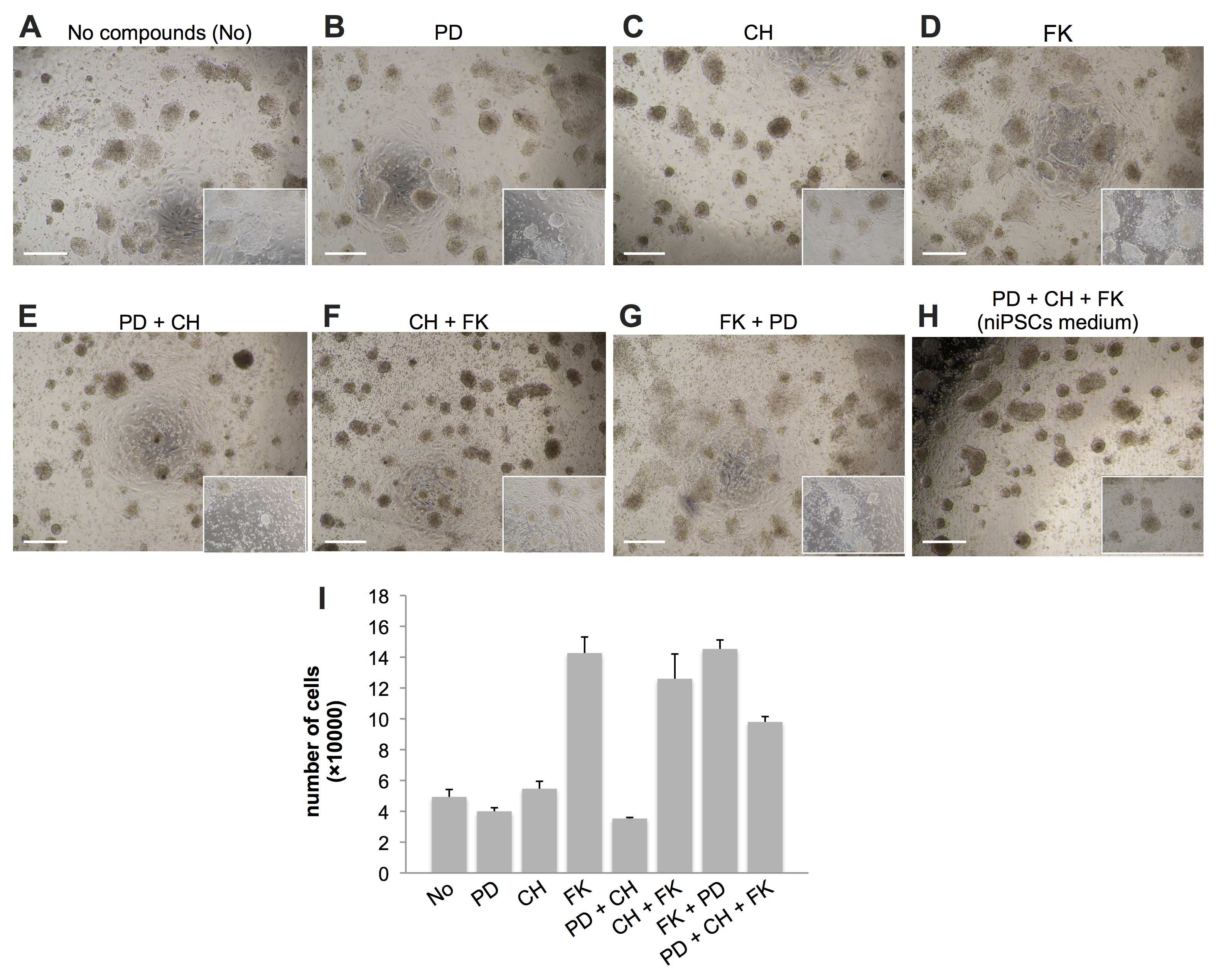

Supplement: S1 Fig — pnbiPSCs cultured only with bLIF (A), bLIF+CH (B), bLIF+PD (C), bLIF+FK (D), LIF+CH+PD (E), bLIF+PD+FK (F), bLIF+FK+CH (G), and bLIF+FK+CH+FK (H). (I) Numbers of growing cells after cultivation in different conditions for 4 days. (A)–(H), scale bars = 500 μm. (TIFF) [file pone.0135403.s001.tiff]

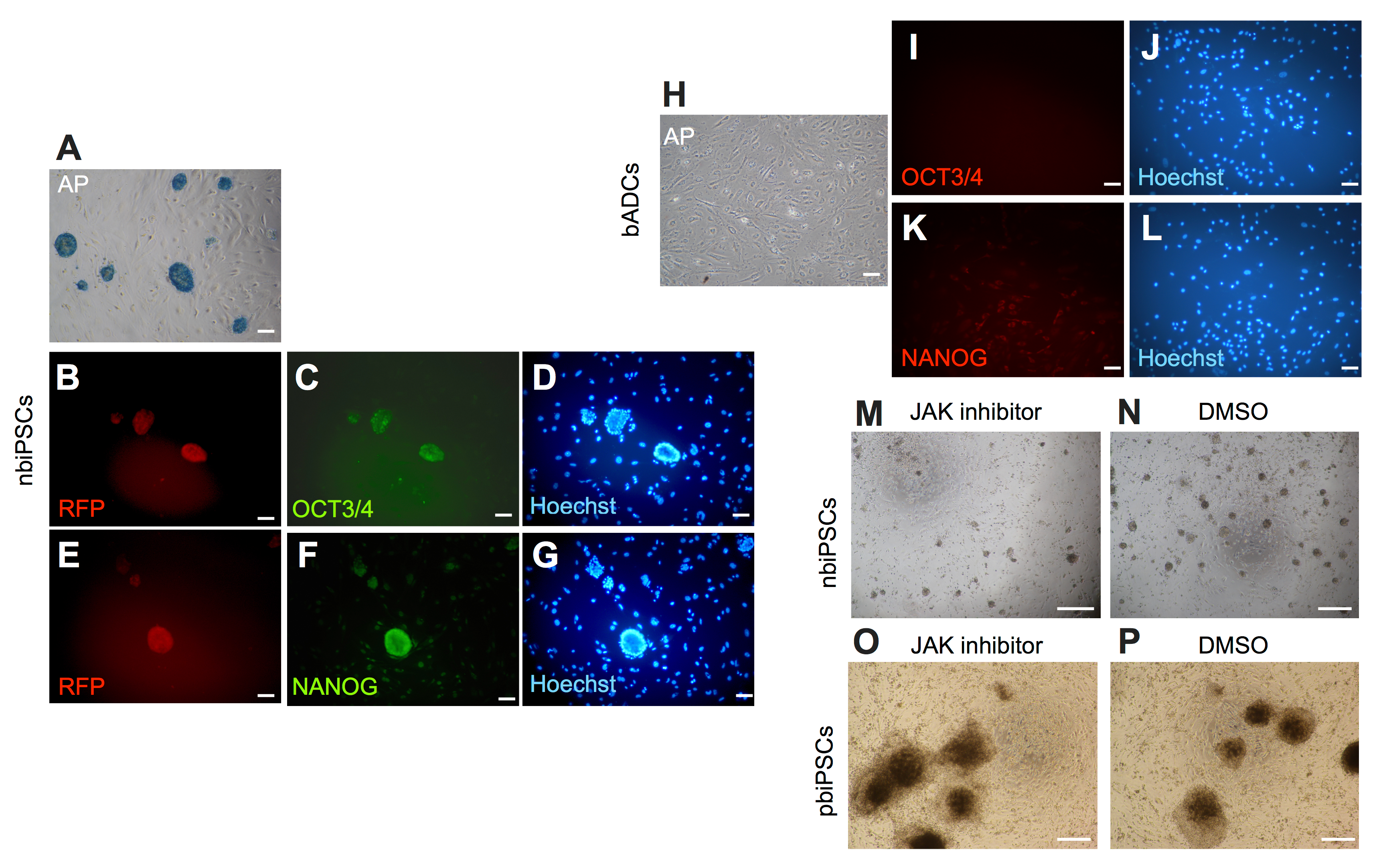

Supplement: S2 Fig — (A) Alkaline phosphatase activity in nbiPSCs. OCT3⁄4 (B, RFP-positive image; C, OCT3⁄4 staining; D, Hoechst staining) and NANOG (E, RFP-positive image; F, NANOG staining; G, Hoechst staining) expression in nbiPSCs expressing RFP. (H) Alkaline phosphatase activity in bADCs. OCT3⁄4 (I, OCT3⁄4 staining; J, Hoechst staining) and NANOG (K, NANOG staining; L, Hoechst staining) expression in bADCs. (M) nbiPSCs cultured in the presence of JAK inhibitor for 4 days. (N) nbiPSCs cultured in the presence of DMSO. (O) pbiPSCs cultured in the presence of JAK inhibitor for 4 days. (P) pbiPSCs cultured in the presence of DMSO. (A)–(L) scale bars = 100 μm. (M)–(P) scale bars = 500 μm. (TIF) [file pone.0135403.s002.tif]

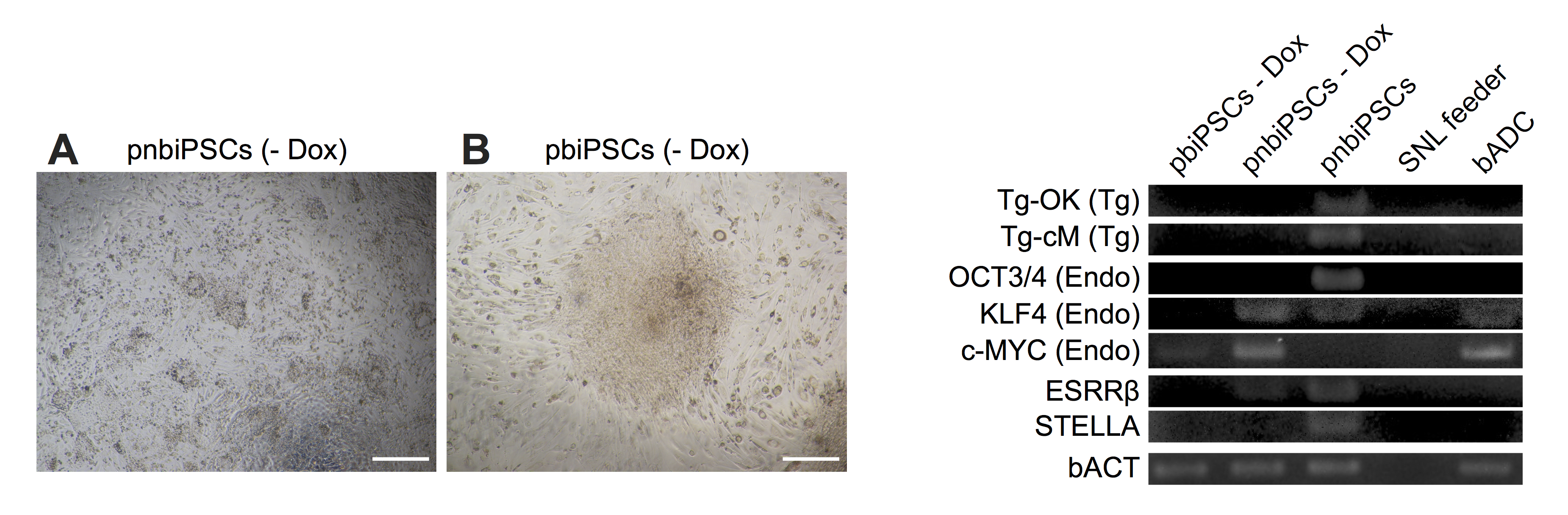

Supplement: S3 Fig — (A) Phase-contrast image of pnbiPSCs cultured in the absence of Dox for 4 days. (B) Phase-contrast image of pbiPSCs cultured in the absence of Dox for 7 days. (C) Endogenous and exogenous gene expression in biPSCs cultured in the absence of Dox. (A), (B), scale bars = 500 μm. (TIFF) [file pone.0135403.s003.tiff]

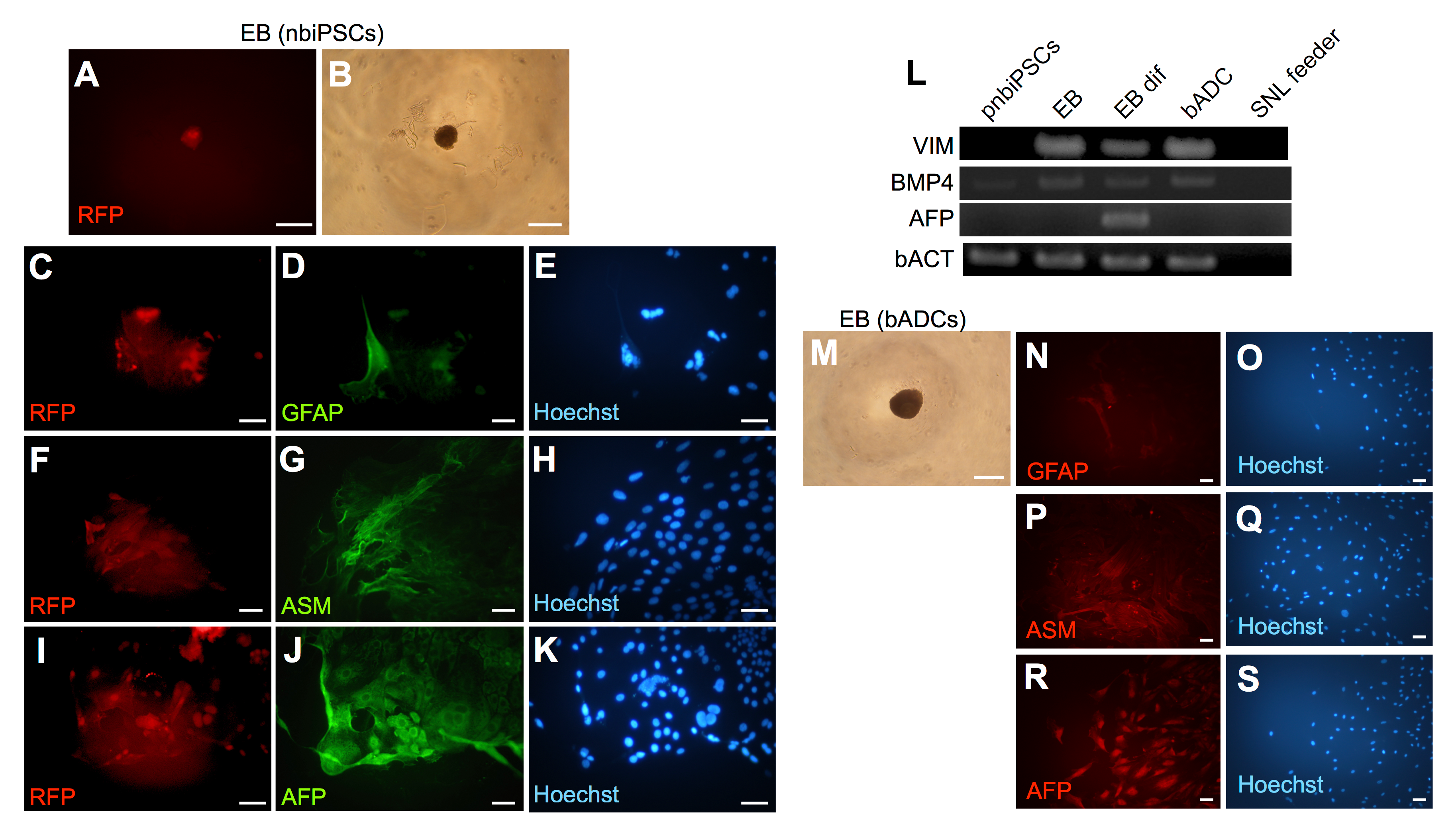

Supplement: S4 Fig — (A) Embryoid body formation of nbiPSCs grown in low cell-adhesion dishes for 6 days. Glial fibrillary acidic protein (C, RFP-positive image; D, glial fibrillary acidic protein; E, Hoechst staining), actin smooth muscle (F, RFP-positive image; G, actin smooth muscle; H, Hoechst staining) and α-fetoprotein (I, RFP-positive image; J, α-fetoprotein; K, Hoechst staining) were used for the markers. (L) Gene-expression profile of pnbiPSCs after embryoid body differentiation. EB, Embryoid body; EB dif, EB-derived cells cultured for an additional 6 days on a gelatin-coated dish; VIM, VIMENTIN. (M) Embryoid body formation of bADCs grown in low cell-adhesion dishes for 6 days. Glial fibrillary acidic protein (N, glial fibrillary acidic protein; O, Hoechst staining), actin smooth muscle (P, actin smooth muscle; Q, Hoechst staining) and α-fetoprotein (R, α-fetoprotein; S, Hoechst staining) were used for the markers. (A), (B), (M) scale bars = 500 μm. (C)–(S) scale bars = 70 μm. (TIF) [file pone.0135403.s004.tif]
